# Single Cell Mechanotype and Associated Molecular Changes in Urothelial Cell Transformation and Progression

**DOI:** 10.3389/fcell.2020.601376

**Published:** 2020-11-19

**Authors:** Weibo Yu, Qing-Yi Lu, Shivani Sharma, Chau Ly, Dino Di Carlo, Amy C. Rowat, Michael LeClaire, Donghyuk Kim, Christine Chow, James K. Gimzewski, Jianyu Rao

**Affiliations:** ^1^Department of Pathology and Laboratory Medicine, University of California, Los Angeles, Los Angeles, CA, United States; ^2^Department of Medicine, University of California, Los Angeles, Los Angeles, CA, United States; ^3^Department of Integrative Biology and Physiology, University of California, Los Angeles, Los Angeles, CA, United States; ^4^Department of Bioengineering, University of California, Los Angeles, Los Angeles, CA, United States; ^5^Department of Chemistry and Biochemistry, University of California, Los Angeles, Los Angeles, CA, United States

**Keywords:** cell mechanotype, bladder cancer, cell stiffness, cell deformability, cancer progression, EMT

## Abstract

Cancer cell mechanotype changes are newly recognized cancer phenotypic events, whereas metastatic cancer cells show decreased cell stiffness and increased deformability relative to normal cells. To further examine how cell mechanotype changes in early stages of cancer transformation and progression, an *in vitro* multi-step human urothelial cell carcinogenic model was used to measure cellular Young’s modulus, deformability, and transit time using single-cell atomic force microscopy, microfluidic-based deformability cytometry, and quantitative deformability cytometry, respectively. Measurable cell mechanotype changes of stiffness, deformability, and cell transit time occur early in the transformation process. As cells progress from normal, to preinvasive, to invasive cells, Young’s modulus of stiffness decreases and deformability increases gradually. These changes were confirmed in three-dimensional cultured microtumor masses and urine exfoliated cells directly from patients. Using gene screening and proteomics approaches, we found that the main molecular pathway implicated in cell mechanotype changes appears to be epithelial to mesenchymal transition.

## Introduction

Urothelial carcinoma (UC) of bladder is the fifth most common cancer in the United States (Siegel et al., 2018). Because of the high rate of disease recurrence and progression, lifelong continued monitoring is an essential part of management, which places a heavy burden on patients and healthcare services (Leal et al., 2016). Urine cytology is the most accessible method to examine potential UC cells. Morphologically, malignant cells present increased nuclear-to-cytoplasmic (N/C) ratio and abnormal nuclear architecture, which provides the basis of cytology diagnosis. However, relying on morphology alone has many limitations. Some high-grade carcinoma cells also have abundant cytoplasm (Renshaw and Gould, 2018). Benign reactive urothelial cells, tissue cluster, viral effect, post-treatment effect, and inflammation could increase the ambiguity and subjectivity. In addition, although in cystoscopy, high-grade invasive carcinoma often appears with large or multiple lesions, there is no specific morphological or molecular features for distinguishing invasive lesions from non-invasive tumors. This has a major clinical implication as invasive tumors, especially muscle invasive tumors, typically require aggressive treatment including radical cystectomy.

Genetically, urothelial bladder cancer has a remarkable propensity for divergent differentiation in association with advanced disease and aggressive behavior and demonstrates heavy mutational burden with an extensive heterogeneity in carcinogenesis (Nordentoft et al., 2014). These changes can be traced for developing molecular diagnostic biomarkers. Currently, FDA has approved NMP22, NMP22 BladderChek, and UroVysion urine assay for bladder cancer diagnosis and surveillance, and immunocytology (uCyt+), BTA-TRAK, and BTA-STAT for surveillance (Santoni et al., 2018). Studies have shown that single or combined molecular tests improve overall sensitivity of cytology to more than 70% (Todenhofer et al., 2013; He et al., 2016). Other DNA-based tests, including Telomerase reverse transcriptase (TERT) mutation assay, DNA methylation, microRNA, and transcriptomic biomarkers exhibited varied sensitivity between 50 and 80% (Beukers et al., 2017; Tan et al., 2018). However, tumor heterogeneity constitutes a main hurdle to the development of robust molecular biomarkers for bladder cancer (Lamy et al., 2016). It is still ambitious to capture the whole complex molecular heterogeneity landscape at cell level for the distinguishing of different malignant stages.

Indeed, the hallmark, and probably the deadliest aspect of cancer, is the invasive and metastatic nature of the disease. Cancer cell invasive and metastatic behaviors are likely the result of altered molecular, biochemical, and biophysical properties that are brought by the complex interplay of activation/inactivation of multiple signaling pathways regulating these cellular events. Recent emerging evidence has indicated that cellular mechanical properties, or mechanotype, is directly relevant to cell malignant phenotype, especially invasion, and metastasis. Our previous study showed that metastatic cancer cells from patients with various types of cancers (lung, breast, and pancreas) are less stiff than benign reactive mesothelial cells from human pleural fluid samples based on Young’s modulus of elasticity determined by atomic force microscopy (AFM) (Cross et al., 2007). The reactive mesothelial cells and metastatic cancer cells often share very similar morphological features, creating difficulties in routine clinical diagnosis. Further studies showed that AFM measurements could be used to predict the response of tumor cells to the treatment of therapeutic drugs (Cross et al., 2008, 2011; Sharma et al., 2012, 2014). In addition, the cancer cell mechanotype evaluated by quantitative deformability cytometry (q-DC) can also be used to predict their invasion across breast and ovarian cancer cell lines (Nyberg et al., 2018). Using microfluidic inertial focusing, hydrodynamic stretching, and high-speed image analysis, we have also demonstrated that cell deformability (i.e., the ability to change shape under load) provides a quantitative marker for objective algorithmic-based diagnoses of malignant pleural effusion cells (Gossett et al., 2012; Tse et al., 2013). Measurements of circulating tumor cells using this technique also revealed a more deformable phenotype than other large cells present in blood (Che et al., 2017). UC of the bladder has a well-defined multi-step nature of development. Urinary exfoliated cells, derived from primary UC tumors, provide a unique living model for the study of UC. However, the cell mechanotype changes of UC cells and urinary exfoliated cells have not previously been systematically studied.

In the present study, we characterized the changes in cellular mechanotype in a well-established multi-step urothelial cancer progression model and clinical urinary specimens using an array of techniques including single cell AFM indentation method, microfluidic-based deformability cytometry (DC) analysis, and q-DC analysis. Gene expression and proteomics analysis were performed to investigate the underlying molecular events associated with malignant phenotype and cell mechanotype changes.

## Materials and Methods

### Cell Culture

A human UC *in vitro* model included HUC-BC, HUC-PC, and MCT-11 cell lines were from the Pathology and Laboratory Medicine Department at the University of California, Los Angeles (UCLA) (Bookland et al., 1992a,b). Cells were grown in Dulbecco’s Modified Eagle’s Medium (DMEM) containing 10% (v/v) fetal bovine serum (FBS) and 1% (v/v) streptomycin/penicillin (S/P), and maintained at 37.0°C with 5% CO_2_. Medium was replaced every 2–3 days depending on cell density. For three-dimensional (3D) cell culture, 2 × 10^3^ cells in a 200 μL DMEM containing 10% FBS were seeded in 96-well spheroid microplates (corning). The plate was incubated for 48 h at 37°C, 5% CO_2_ to allow the formation of cell spheroid. Cultured HUC-BC, HUC-PC, and MCT-11 cells in 50% confluency were treated with 200 μM 4-ABP or 60 μg/mL GTE (both from Sigma-Aldrich), which were determined by cell proliferation assay. Cells were exposed for 48 h prior to harvesting for mechanotype analysis.

### Cell ImageStream Morphology Analysis

We used the ImageStreamx MarkII imaging flow cytometer to discriminate subtle morphologic or signal distribution changes within cell populations. Treated and untreated HUC cell suspensions with a concentration of 2 × 10^7^ cells/mL in PBS/2%FBS were labeled with Texas red and DAPI. For each cell, a side-scatter (darkfield) image, a transmitted light (brightfield) image, and two fluorescence images of G-actin and nuclear DNA were acquired to analyze the changes of cell diameter and nuclear area.

### Urinary Specimen Collection and Processing

Urinary exfoliated cells were collected from a 20 mL urinary specimen after centrifugation and then attached on slides through cytospinning at 100 rpm for 5 min. We previously used short-term *ex vivo* culture to allow cell attachment (Cross et al., 2007), but the culturing step is time consuming and introduces artifacts. The cytospin method is fast and preserves the morphology of urine cells well, which has been verified in our laboratory. Cytospun cells were covered with DMEM/F-12 medium, scanned under 200X microscope field, and measured Young’s modulus on uroepithelial cells, which can be distinguished from squamous epithelial cells and cells of hematologic origin.

### Analysis of Cell Young’s Modulus Using AFM

Treated and untreated HUC-BC, HUC-PC, and MCT-11 cells (1 × 10^5^ cell/mL) were seeded in 60 × 15 mm petri dishes. AFM measurement was performed when cells completely attached on the surface using a Catalyst Bioscope (Bruker) with a combined inverted optical/confocal microscope (Zeiss). This combination permits lateral positioning of the AFM tip over the nuclear region of the cell with micrometer to nanometer precision. Mechanical measurements were carried out at 37°C using silicon nitride cantilevers with experimentally determined spring constants. Force–displacement curves were recorded at 1 KHz for determination of Young’s modulus. The modulus was calculated by converting the force curves into force–indentation curves and fitting with the Hertz–Sneddon model, which describes the indentation of an elastic sample using a stiff conical indenter on cell nuclear area. To prevent damage to the cell surface and to reduce any possible substrate-induced effects, measurements were performed in force ranges resulting in shallow indentations of the cell (< 400 nm). We measured about 15 cells in each sample. Data were plotted as histograms of Young’s modulus (E, KPa) vs. relative frequency for each measured sample.

### Analysis of Cell Deformability Using DC

Treated and untreated HUC-BC, HUC-PC, and MCT-11 were detached and suspended at 1 × 10^5^ cells/mL for DC measurement. Microfluidic devices were fabricated using standard photolithographic methods and polydimethylsiloxane (PDMS) replica molding techniques. Cell suspensions were pumped through PEEK tubing inserted into the DC microfluidic chip by a syringe pump with a volumetric flow rate ranging from 700 to 1,075 μL/min to test various stresses on cell response. High-speed (350,000 frames/s) video was acquired, and an automated image analysis algorithm was used to extract cell size and shape metrics. Automated image analysis software was used to extract a host of independent physical parameters from these images. The software stores and graphs strain metrics (deformability = a/b, where a is the long axis dimension of the cell and b is the short axis) properties of 1,000 of cells in a density scatter plot format.

### Analysis of Cell Transit Time Using q-DC

Monolayer cells in culture were detached and suspended at 1 × 10^5^ cells/mL for q-DC experiment. Q-DC microfluidic devices were mounted onto an inverted microscope (Zeiss Observer, Zeiss, Oberkochen, Germany) that was equipped with a 20/0.40 NA objective. Cell suspensions were driven by a constant air pressure (69 kPa) to flow through the channels. As cells deformed through microfluidic constrictions with 7 μm height and 7 μm width, a CMOS camera (MicroRNAcoEx4, Vision Research, Wayne, NJ, United States) was used to capture brightfield images at rates of 600–2,000 frames per second. Using custom software^1^ (MATLAB), we analyzed the displacements of single cells through the microfluidic channel and extracted transit time based on the time a cell entered and exited a constriction (Nyberg et al., 2016).

### Gene Expression Analysis

Total RNA was extracted from treated and untreated cells using a TRIzol reagent (Thermo Fisher Scientific, United States). The RNA samples were then applied to a RNeasy Mini spin column for purification (RNeasy Miniprep Kit, Qiagen). Global gene expression profiles of the cells were generated using the Affymetrix GeneChip Human Transcriptome Array (HTA) 2.0 system. An Affymetrix WT PLUS Reagent Kit was used to prepare the RNA for hybridization to the HTA. Data analysis was performed using the Affymetrix Expression Console software and Transcriptome Analysis Console software. Ingenuity Pathway Analysis (IPA) was followed to compare the changes in gene expression in different signaling pathways.

### Quantitative Analysis of Protein Level of Urothelial Cells by Mass Spectrometry

Peptide sample preparation, LC-MS acquisition, and analysis were carried out at the UCLA Proteome Research Center. Briefly, protein aliquots (50 μg) were reduced and alkylated via sequential incubations of 5 mM tris(2-carboxyethyl)phosphine hydrochloride and 10 mM iodoacetamide at room temperature, allowed to bound to beads after the addition of 10 μL of carboxylate-modified magnetic beads (Hughes et al., 2014), and then digested by sequential addition of lys-C and trypsin proteases. Peptide samples were separated on a 75 μm i.d., 25 cm C18 column packed with 1.9 μm C18 particles (Dr. Maisch GmbH HPLC) using a gradient of increasing acetonitrile concentration and injected into an Orbitrap-Fusion Lumos Tribrid mass spectrometer (Thermo Fisher Scientific, United States), on which MS/MS spectra were acquired by Data Dependent Acquisition (DDA) mode. Database searching was performed using the MaxQuant (1.6.10.43) against the human reference proteome from EMBL (UP000005640_9606 HUMAN Homo sapiens, 20,874 entries). The search included carbamidomethylation on cysteine as a fixed modification and methionine oxidation and N-terminal acetylation as variable modifications. The digestion mode was set to trypsin and allowed a maximum of two missed cleavages. The precursor mass tolerances were to 20 and 4.5 ppm for the first and second searches, respectively, while a 20 ppm mass tolerance was used for fragment ions. Datasets were filtered at 1% FDR at both the PSM and protein-level. Peptide quantitation was performed using the MaxQuant’s LFQ mode.

### Immunohistochemical Staining Analysis

Cells were harvested, washed, and then fixed to prepare for Immunohistochemical staining (IHC) analysis. Cells were placed on slides through the cytospin procedure. We used Citrate buffer PH 6.0 to perform antigen retrieval. The antibodies used were M0725 anti-vimentin IgG (Dako), and pan-cytokeratin (AE1/AE3) (Cell Marque) mouse monoclonal antibody with a concentration of 1/100. We incubated cell slides with the first antibody at 4°C overnight. Then second antibody incubation and DAB process were performed to develop stain color.

### Statistical Analysis

Statistical analysis was performed using the SAS version 9.2 software (SAS Institute Inc., Cary, NC, United States). Continuous data were presented as means plus/minus the standard deviation (± SD) and compared with the Student’s *t*-test and one-way analysis of variance. For cell transit time, we used the Mann-Whitney U test to determine statistical significance. Categorical variables were compared by the chi-square (χ^2^) test. Statistical significance was defined by a two-tailed *p*-value of ≤ 0.05.

## Results

### Characterization of Urothelial Cell Mechanotype During Malignant Transformation and Progression Using AFM, DC, and q-DC

UC, like many other epithelial malignancies, typically follows a multi-step development and progression from normal to non-invasive *in situ* to invasive carcinoma (Humphrey et al., 2016; Wang and Mckenney, 2019). To recapitulate the multi-step nature, we used a unique carcinogenic human urothelial carcinoma (HUC) model. The HUC model includes three cell lines. These cell lines, derived originally from the same progenitor cells, undergo different malignant transformation, and show distinct progression potentials in response to the treatment of carcinogen 4-aminobiphenyl (4-ABP) (Bookland et al., 1992a,b). The HUC-BC is untransformed cells and not tumorigenic (hence simulating “normal urothelial cells”), the HUC-PC exhibits non-invasive transformation phenotype (simulating “preinvasive” urothelial cells), and the MCT-11 is transformed carcinoma cells and exhibits typical invasive and aggressive phenotype.

**FIGURE 1:**
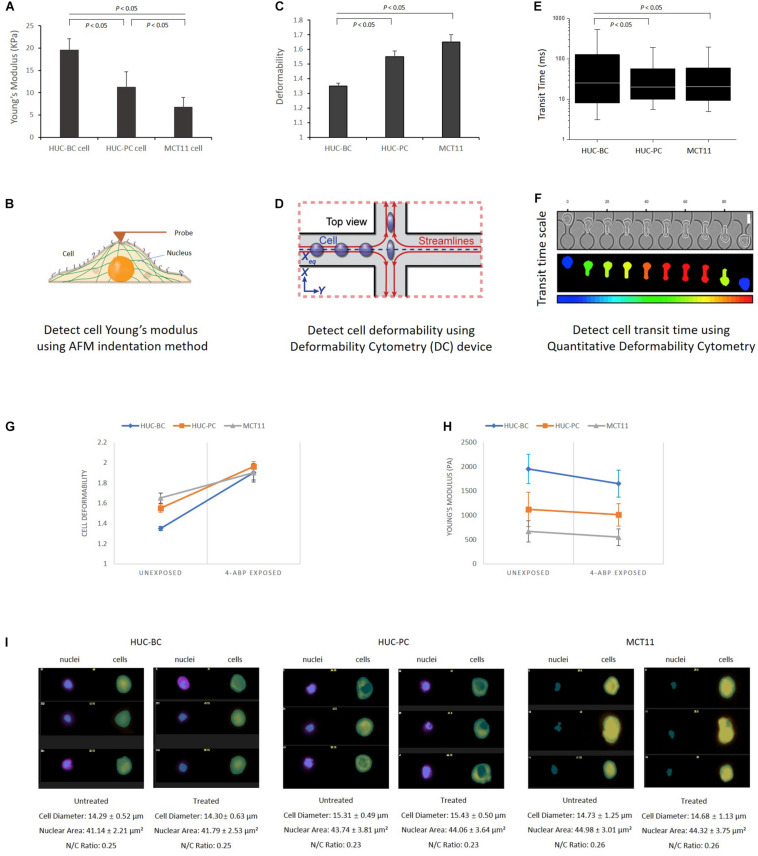
Characterization of urothelial cell mechanotypes using AFM, DC, and q-DC techniques. **(A,B)** Young’s Moduli of HUC-BC, HUC-PC, and MCT-11 cells were characterized by AFM. Force–displacement curves generated by probe over the nuclear region were recorded for determination of Young’s moduli of HUC cells. **(C,D)** Deformability of HUC-BC, HUC-PC, and MCT-11 cells was analyzed using DC device. Cell deformation is induced by the shear stresses of fluid flow and captured by a high-speed imaging system. An automated image analysis algorithm was used to extract cell size and shape metrics. Cell maximum aspect ratio under compressive and shear forces was defined as deformability. **(E,F)** Transit time of HUC-BC, HUC-PC, and MCT-11 cells was analyzed using q-DC device. The displacements of single cells through the microfluidic channel were recorded. Transit time based on the time a cell entered and exited a constriction was extracted. **(G,H)** Decreased cellular Young’s modulus and increased deformability were observed in HUC-BC, HUC-PC, and MCT-11 cells after 48 h carcinogen 4-ABP exposure through AFM and DC analysis. **(I)** Transmitted light (brightfield) image and nuclear images of HUC-BC, HUC-PC, and MCT-11 cells are shown by the ImageStream flow cytometry. Cell sizes, nuclear areas, and cell/nucleus ratio between exposed and unexposed cells were quantified. AFM, atomic force microscope; DC, deformability cytometry; q-DC, quantitative deformability cytometry; HUC, human urothelial carcinoma.

To investigate the cellular mechanotype changes, three previously established platforms were used (Cross et al., 2007; Gossett et al., 2012; Nyberg et al., 2017). As shown in Figures 1A,B, AFM characterizes elasticity, or stiffness, of living cells through measuring force–displacement as the tip is pushed toward the cell, indented into the sample, and subsequently retracted (Cross et al., 2007). The stiffness of cells is quantified by the value of Young’s modulus. From untransformed HUC-BC cells, HUC-PC cells to transformed MCT-11 cells, the stiffness gradually decreases (HUC-BC vs. HUC-PC, cell modulus: 19.5 ± 2.6 KPa vs. 11.2 ± 4.5 KPa, *P* < 0.05, *t*-test; HUC-BC vs. MCT-11, cell modulus: 19.5 ± 2.6 KPa vs. 6.7 ± 2.2 KPa, *P* < 0.05, *t*-test) (Figure 1A). The inversely related trend was observed in cell deformability measurement by DC in which a continuous stream of single cells is created, where each cell’s deformation under a high-speed microfluidic flow is measured with high-speed imaging (Gossett et al., 2012). DC enables high-throughput single-cell mechanotyping. Cellullar deformation is induced by the shear and inertial stresses of fluid flow (Figure 1D). DC measurements show that in the order of HUC-BC, HUC-PC, and MCT-11 cells, deformability progressively increases (HUC-BC vs. HUC-PC, deformability: 1.35 ± 0.02 vs. 1.55 ± 0.04, *P* < 0.05, *t*-test; HUC-BC vs. MCY-11, deformability: 1.35 ± 0.02 vs. 1.65 ± 0.05, *P* < 0.05, *t*-test) (Figure 1C). To confirm this finding, we analyzed cellular deformability using another technique, q-DC (Nyberg et al., 2017). By applying a pressure gradient across the microfluidic device, cells were driven to deform through micron-scale constrictions (Figure 1F). We tracked the time scale for single cells to transit through micron-scale gaps in the microfluidic channel; the resultant “transit time” reflects cell mechanotype, whereby stiffer cells and particles have a longer transit time (Nyberg et al., 2016). In the pooled dataset that includes three replicates, HUC-PC and MCT-11 cells showed a statistically significant decrease in transit time compared to HUC-BC cells (HUC-BC vs. HUC-PC, median transit time: 25.6 vs. 20.0 ms, *P* < 0.05, U-test; HUC-BC vs. MCT-11, median transit time: 25.6 vs. 20.6 ms, *P* < 0.05, U-test), substantiating an increase in the compliance of highly malignant UC cells compared to the untransformed urothelial cells (Figure 1E). These results indicate that highly malignant carcinoma cells are less stiff and more deformable than untransformed cells.

### Decreased Stiffness in HUC Cells From 3D Cultured Microtumor Mass and From Patient Urinary Cytology Specimens

**FIGURE 2:**
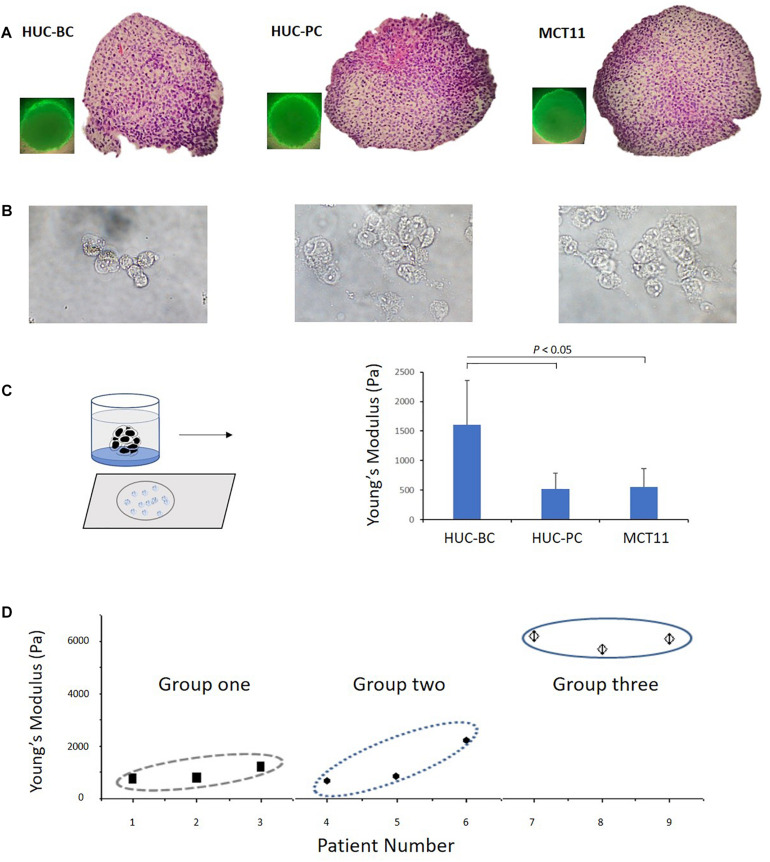
Change in Young’s modulus of cells from 3D cultured microtumor masses and from patient urinary cytology specimens. **(A)** A 3D cell culture condition was used to grow HUC-BC, HUC-PC, MCT-11 cells as micro tumor masses. Spheroid formations of HUC cells were shown over 72 h after seeding. HE stains demonstrate dense, rapid-growing cell masses. **(B)** Dissociated HUC-BC, HUC-PC, and MCT-11 cells were under AFM measuring. **(C)** Untransformed HUC-BC cells display significant higher cellular Young’s modulus than HUC-PC and MCT-11 cells. **(D)** Pooled data analysis of Young’s moduli of urinary exfoliated cells from nine urine cytology specimens. Group one included three cytology-positive specimens from patients with HGUC confirmed by cystoscopy biopsy diagnosis. Group two included thee cytology-atypical specimens from patients with HGUC confirmed by cystoscopy biopsy diagnosis. Group three included three cytology-negative specimens from patients with no UC. The number of measured uroepithelial cells in each case ranged from 3 to 10. The average number of force-displacement curve obtained from each cell was three. AFM, atomic force microscope; HUC, human urothelial carcinoma.

To better mimic *in vivo* tumor growth, we used a scaffold-free spheroid cell culture condition to grow bladder cancer cells as micro tumor masses (Figure 2A). Prior to AFM measurement, cells were dissociated from tumor masses and placed on the slides after cytospin (Figure 2B). Again, we observed that untransformed HUC-BC cells display significantly higher stiffness than HUC-PC and MCT-11 cells (Figure 2C).

Based on our previously published methods (Cross et al., 2007; Gossett et al., 2012), we developed a practical protocol to prepare cells directly from urinary cytology specimens for mechanotype analyses described in the section “Materials and Methods”. Using this method, we tested nine clinical urinary specimens, which were divided into three groups. Group one and two included six specimens from patients with high-grade urothelial carcinoma (HGUC) confirmed by cystoscopy biopsy diagnosis. The three specimens of group one had positive cytology findings and the three specimens of group two had atypical cytology findings. Group three included another three from patients with no UC as confirmed by both cystoscopy and cytology examination. As shown in Figure 2D, a pooled data analysis demonstrated the same mechanotype change pattern as we observed in 3D cultured microtumor masses, where urinary exfoliated cells from group three had the highest Young’s modulus. While certainly more samples will be needed to be conclusive, the preliminary finding shows the feasibility of measuring single-cell mechanotype changes in clinical urinary samples.

### Decreasing Stiffness and Increasing Deformability During Malignant Transformation and Progression Induced by 4-ABP

As shown in Figure 2C, the magnitude of difference between HUC-BC vs. HUC-PC and MCT-11 cells cultured from 3D condition appeared to be larger than the differences in cellular stiffness measured by AFM on 2D cultured cells. We suspected that in microtumor masses, HUC cells were grown without losing cell-cell interaction in all directions, central hypoxia, and cell response and resistance to the tumor microenvironment. The malignant potential can be fully expressed and captured by mechanotype analysis. Previously, it has been shown that upon exposure to carcinogen 4-ABP, the malignant phenotypes, namely the ability of cells to form tumor nodules upon injected to the nude mice, can be enhanced (Bookland et al., 1992a,b). Thus, to further characterize the link between mechanotype change and urothelial transformation and progression, we exposed three cell lines to 4-ABP. After 48-h exposure, we quantified morphological changes of cells using the ImageStream flow cytometry analysis. No significant differences were observed in cell sizes, nuclear areas, and cell/nucleus ratio between treated and untreated cells (Figure 1I). However, decreased cell Young’s modulus was observed in HUC-BC, HUC-PC, and MCT-11 cells through AFM measurement (Figure 1H). A similar trend in the changes of cell deformability was seen by DC, whereby all three cell lines displayed increased deformability compared to untreated controls (Figure 1G). Altogether, mechanotype characterization of the urothelial cells demonstrated specifically decreased cellular modulus and increased cell deformability as malignancy increases, indicating that changes in cellular mechanotype is associated with urothelial cell malignant transformation and progression.

### Correlation of Activated Epithelial-Mesenchymal Transition Pathway With the Change of Cellular Mechanotype

**FIGURE 3:**
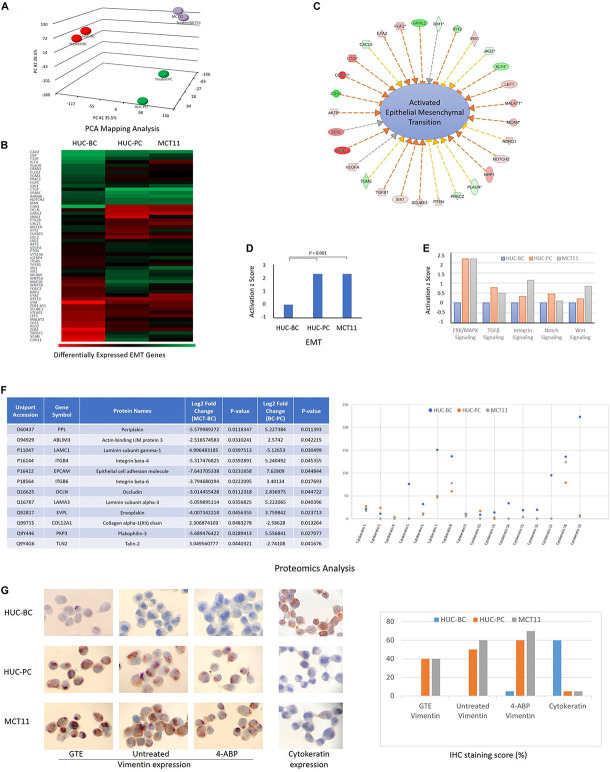
Molecular events associated with mechanotype changes of urothelial cells. **(A)** PCA mapping analysis shows the differences in gene expression among HUC-BC, HUC-PC, MCT-11 cells, and their 4-ABP exposed counterparts. **(B–D)** demonstrate the activated EMT process from HUC-BC, HUC-PC, to MCT-11 cells (IPA analysis). **(E)** Shows activated ERK/MAPK, TGFβ, Integrin, Notch, and Wnt signaling from HUC-BC, HUC-PC, to MCT-11 cells. **(F)** Proteomics analysis identifies differentially expressed proteins associated with cell mesenchymal and epithelial features. Protein markers that had more than twofold expression changes between MCT-11 and HUC-BC, and between HUC-BC and HUC-PC are listed in the right table. Individual cytokeratin expression is shown on the right. **(G)** Vimentin expression on untreated, GTE-treated, and 4-ABP-treated cells and pan-cytokeratin expression on HUC model cells are shown on IHC staining pictures. Staining score was reported as the ratio number of positively stained cells/total number of cells × 100. PCA, Principal component analysis; IPA, Ingenuity pathway analysis; GTE, green tea extract.

**FIGURE 4:**
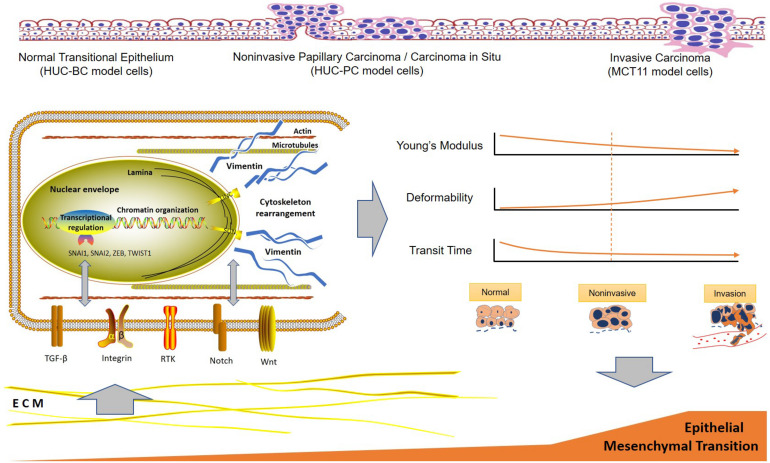
Hypothetical scheme of the interactions between urothelial cell mechanotype change and epithelial to mesenchymal transition process during malignant transformation and progression. ECM, extracellular matrix.

To explore the underlying molecular events associated with these specific mechanotype changes, we screened the transcriptome of HUC-BC, HUC-PC, and MCT-11 cells. The 3D portrayal diagram (Figure 3A) demonstrates the differences in gene expression among the three cell lines and their carcinogen-induced counterparts. Using IPA, we identified the association of many differentially expressed genes with epithelial to mesenchymal transition (EMT). Compared to HUC-BC cells, HUC-PC, and MCT-11 cells have more than two fold activation in the EMT pathway (activation *z* score: 2.319, *P* < 0.001) (Figures 3B–D). Upstream regulator analysis indicated several molecular signaling pathways, including ERK/MAPK, TGFβ, Integrin, Notch, and Wnt signaling, all of which are important contributors of the EMT pathway, were activated (Figure 3E). The EMT process was originally found in tissue repair and fibrosis, in which epithelial cells lose their junctions and apical-basal polarity and motile behavior as they differentiate into mesenchymal cells. Importantly, the EMT process involves gene expression reprogramming, signaling changes and cytoskeleton reorganization, and plays a pivotal role in malignancy progression (Sarrio et al., 2008; Thiery et al., 2009). Recent genome-wide characterization study on 112 bladder cancer cases revealed that highly malignant sarcomatoid urothelial bladder cancer is largely driven by dysregulation of the EMT network (Guo et al., 2019). However, the evidence of a full EMT phenotype in clinical carcinomas and metastases is frequently questioned because EMT can be reversible and transient (Brabletz, 2012). Also, the role of the EMT process as a driving force on cell mechanotype regulation is not clear. To confirm the change between activated EMT and decreased cell’s modulus and increased deformability, we induced malignant progression on HUC-BC, HUC-PC, and MCT-11 cells with 4-ABP. As shown in Figures 1G,H, 4-ABP exposure reduced Young’s modulus and increased deformability in all three cell lines, especially in HUC-BC and HUC-PC cells. We then performed an IPA canonical pathway analysis on differentially expressed genes. Again, activated EMT pathway was found in 4-ABP treated HUC-BC and HUC-PC cells in comparison to their untreated counterparts (HUC-BC vs. treated HUC-BC: Activation *z* score: 0.1, *P* < 0.001; HUC-PC vs. treated HUC-PC: Activation *z* score 0.08, *P* < 0.001). These results suggest that the EMT pathway may be correlated with mechanotype dynamics in urothelial cells.

### Identifying Protein Targets in Epithelial-Mesenchymal Transition Pathway Associated With Mechanotype Changes

We then interrogated the differences in protein content using the proteomics approach (Michalski et al., 2011). A number of differentially expressed proteins associated with EMT, including peiplakin, a component of desmosomes and of the epidermal cornified envelope in keratinocytes, E cadherin, an epithelial marker localized at cell–cell contacts, mesenchymal marker vimentin, fibronectin, and many other regulator proteins (Kokkinos et al., 2007) were identified. The table of Figure 3F lists a few protein markers that had more than twofold expression changes between MCT-11 and HUC-BC, and between HUC-BC and HUC-PC. The change trends of both epithelial marks and mesenchymal markers demonstrated urothelial cells obtained mesenchymal features while losing epithelial makers with malignant progression, which is consistent with the results from gene expression analysis.

Cytokeratins are a class of intermediate filaments demonstrating epithelial differentiation. Individual cytokeratins are commonly used markers for determining the grade of bladder cancer cells (Kim et al., 2015; Hashmi et al., 2018). As illustrated in Figure 3F, protein abundance of individual cytokeratins showed the expression of most cytokeratin family molecules decrease from HUC-BC, to HUC-PC, and MCT-11 cells. Among them, cytokeratin 5, 7, 17, and 19 have remarkable decreasing trend. This trend was confirmed by pan-cytokeratin IHC staining, where HUC-BC cells demonstrated significantly higher pan-cytokeratin expression than HUC-PC and MCT-11 cells (Figure 3G). On the other hand, vimentin, an EMT marker, is one of the intermediate filaments that mainly functions to maintain cell integrity and involved in the cell migration and invasion of metastasizing cancer cells (Coulombe and Wong, 2004; Mendez et al., 2010). Our previous study showed that ovarian cancer cells induced to have mesenchymal phenotype either by overexpression of EMT transcription factors (ZEB1, SNAI1, and SNAI2), oncogenes (H-Ras v12), or by drug resistance were all consistently more deformable than cells with epithelial phenotype (Qi et al., 2015). In these processes, increased vimentin and reduced E-Cadherin were indicated. Over-expression of vimentin correlates with increased tumor growth and invasiveness, and as well as with poor clinical outcomes in several cancers (Maier et al., 2016). Another study reported that intracellular mechanical homeostasis was interrupted in vimentin-knockdown breast cancer cells. Overexpressing vimentin in MCF7 breast cancer cells reoriented microtubule polarity, increased cell directional migration, and EMT phenotypes (Liu et al., 2015). To confirm the upregulation of vimentin in UC cells, we performed an IHC analysis. HUC-BC cells have the lowest expression of vimentin whereas MCT-11 cells have the highest, in line with the activation status of EMT and urothelial cell mechanotype changes (Figure 3G). Previously, we also reported that chemopreventive agent green tea extract (GTE) modulated cytoskeletal actin remodeling via Rho activity in the same UC cells and significantly increased the stiffness of GTE-treated metastatic tumor cells compared to normal cells (Lu et al., 2005; Cross et al., 2011). In the present study, we found that GTE counteracts the effect of carcinogen 4-ABP by reversing the increase in vimentin expression (Figure 3G), further corroborating epithelial-mesenchymal transition plays an important role in urothelial cell mechanotype changes.

## Discussion

While metastatic tumor cells show a distinctive cell mechanotype relative to normal cells, the specific pattern of changes in cell mechanotype during the early stages of cancer transformation and progression is not well studied. Identifying metastatic cancer cells may be clinically too late to save patients’ lives. Therefore, there is a great need for developing biomarkers that can be used to determine the invasive and metastatic potential of malignant cells before the invasion or metastasis actually occur. Moreover, the molecular mechanism underlying the changes in cellular mechanotype is poorly studied and understood. Recent evidence suggests that the regulation of cellular mechanotype may be the result of alterations of multiple genes and signal transcription pathways (Way and Weeds, 1990; Provenzano et al., 2009; Lammerding, 2011; De Las Heras et al., 2013; Fedorchak et al., 2014). Thus, knowledge of cell mechanotype changes in the early stages of cancer transformation and progression, and the mechanism or molecular pathways associated with these biomechanical changes may have a significant impact on not only developing biomarkers that can be utilized to distinguish invasive cancer, but also finding drug targets for disrupting, or inhibiting cancer cell invasion or metastasis.

We, here, report the cell mechanotype profiles in relation to UC utilizing an *in vitro* human urothelial carcinogenesis model that recapitulates the multistep process of cancer progression. Cellular Young’s modulus and deformability were analyzed using AFM indentation, microfluidic-based DC, and q-DC analyses. From normal, to preinvasive, to invasive cells, Young’s modulus, or cell stiffness, progressively decreases. Cellular deformability significantly increases. Previous studies indicated that changes in cell mechanotype could be detected in precancerous cervical intraepithelial lesions as early as CIN II (Ding et al., 2015), and also in precancerous esophageal cells (Fuhrmann et al., 2011). Our malignant transformation/progression experiments implied that cellular mechanotype changes may start at the early stage of malignant progression. To verify this finding in a disease-specific setting, we integrated cell mechanotyping technology into clinical samples. Currently, a variety of platforms have been developed to measure cellular mechanical properties, and different techniques are known to probe mechanical properties at different timescales and depths of the cell (Urbanska et al., 2020). The most commonly explored method is to use biomechanical probes, represented by AFM and magnetic tweezer (Yang et al., 2011), whereas cell deformability can be optically probed under the force induced on the whole cells. These methods also include micropipette aspiration (Oh et al., 2012), microfluidic assays (Guo et al., 2014), DC (Gossett et al., 2012), and microplate stretcher (Thoumine et al., 1999). More recently, high-throughput techniques have also been explored (Darling and Di Carlo, 2015). Most of these techniques were developed in a laboratory setting and are still in a pre-clinical stage. Urinary exfoliated cells, which are directly from the primary tumor, provide a unique living model for the study of UC. However, for clinical specimens, variability is common in terms of cell number and type. Under AFM, uroepithelial cells, squamous epithelial cells, and cell of hematologic origin can be easily distinguished. Although only nine specimens were preliminarily analyzed in the current study, the findings were consistent with those from *in vitro* condition. Additionally, it presented a proof of concept for the development of mechanotype signature for urothelial cancer early detection.

It has become increasingly clear that EMT is driven by at least four fundamental regulatory network layers. The first layer is EMT-inducing transcription factor control, such as SNAI1, ZEB, and TWIST1. Another three layers are the expression of small non-coding RNAs, differential splicing, and translational and post-translational control, which determine the stabilization and localization of network proteins (De Craene and Berx, 2013). The dynamic and transient nature of EMT comprising a broad spectrum of intermediate phenotypes adds further complexity to capture EMT molecular status to determine the diagnosis and progression of cancer (Santamaria et al., 2017). Our findings indicate a close correlation between EMT and cell mechanotype in the process of malignant progression, which provides new insight for developing novel biomarkers for cell-based UC early detection. The underlying molecular events and the specific mechanotype changes during UC development and progression can be summarized in Figure 4. One may expect that progression of EMT drives cell mechanotype changes during malignant transformation and progression. Conversely, it is also possible that change in cell mechanotype, such as decreasing in cell stiffness and increasing in deformability that occurs during malignant progression, enables tumor cells to detach from a primary tumor, squeeze through confined spaces, and facilitate cells to invade the surrounding tissue that affects EMT.

In summary, we described the specific mechanotype changes of urothelial cells in malignant transformation and progression. Measurable cell mechanotype changes of stiffness, deformability, and cell transit time occur early in the transformation process. As cells progress from normal, to preinvasive, to invasive cancer cells, Young’s modulus of stiffness decreases and deformability increases gradually. The key molecular pathway implicated in urothelial cell mechanotype changes appears to be associated with EMT.

## Data Availability Statement

The datasets presented in this study can be found in online repositories. The names of the repository/repositories and accession number(s) can be found below: http://www.ebi.ac.uk/arrayexpress/experiments/E-MTAB-9699, accession no: E-MTAB-9699.

## Ethics Statement

The studies involving human participants were reviewed and approved by UCLA-MIRB2. Written informed consent for participation was not required for this study in accordance with the national legislation and the institutional requirements.

## Author Contributions

WY: cell experiment, clinical sample processing, gene expression analysis, protein expression experiment, and manuscript writing. Q-YL: proteomics analysis, data interpretation, manuscript critical review, and revision. SS: AFM experiment, study design, data analysis, and manuscript revision. CL: qDC experiment. DD: DC cell deformability analysis and manuscript revision. AR: qDC analysis and manuscript revision. ML: AFM experiment. DK: DC experiment. CC: manuscript review. JG: study design and AFM cell stiffness analysis. JR: study overall conception and design, data analysis and interpretation. All authors contributed to the article and approved the submitted version.

## Conflict of Interest

The authors declare that the research was conducted in the absence of any commercial or financial relationships that could be construed as a potential conflict of interest.

## Footnotes

1 https://github.com/rowatlab
